# First molecular evidence of *Hepatocystis* infection in non-human primates from Indonesia using fecal DNA: Implications for wildlife surveillance and One Health

**DOI:** 10.14202/vetworld.2025.3651-3669

**Published:** 2025-11-29

**Authors:** Firmanul Hasan, Josephine Elizabeth Siregar, Normalita Eka Pravitasari, Andita Fitri Mutiara Rizki, Wihda Aisarul Azmi, I Made Artika, Wanda Kuswanda

**Affiliations:** 1 Department of Biochemistry, Faculty of Mathematics and Natural Sciences, IPB University, Dramaga Campus, Bogor 16680, Indonesia; 2 Eijkman Research Center for Molecular Biology, National Research and Innovation Agency, Cibinong, Bogor 16911, Indonesia; 3 School of Tropical Medicine and Global Health, Nagasaki University, Nagasaki City, Japan; 4 Research Center for Biota Systems, National Research and Innovation Agency, Cibinong, Bogor 16911, Indonesia

**Keywords:** fecal DNA, *Hepatocystis*, Indonesia, non-human primates, One Health, *Plasmodium*, small subunit ribosomal RNA

## Abstract

**Background and Aim:**

*Hepatocystis*, a malaria-like hemoparasite closely related to *Plasmodium*, infects non-human primates (NHPs), bats, and other mammals, yet remains understudied in Indonesia. Although *Plasmodium* detection in primates has been extensively reported, molecular confirmation of *Hepatocystis* in Indonesian wildlife is lacking. This study aimed to screen NHP fecal samples for *Plasmodium* infection and to identify any malaria-like parasites using molecular methods.

**Materials and Methods:**

A total of 227 fecal samples from captive and rescued NHPs, representing multiple *Macaca* species and other primates, were collected from Tasikoki Wildlife Rescue Center, Manado, Indonesia, in 2019 and 2021. Genomic DNA was extracted using a QIAamp Fast DNA Stool Mini Kit (Qiagen, Germany) and screened for *Plasmodium* using polymerase chain reaction (PCR) targeting the mitochondrial *small subunit ribosomal RNA* gene. Positive amplicons were purified, sequenced, and analyzed using the basic local alignment search tool and phylogenetic reconstruction with MEGA X.

**Results:**

Eight (3.5%) of 227 samples yielded positive PCR bands of approximately 600 bp, differing from the expected 467 bp for *Plasmodium*. Sequencing of four representative samples (MNig-01, MNig-17, MNig-18, and HM-160) revealed >99.7% identity with *Hepatocystis* spp. (GenBank: KY653782.1). Multiple sequence alignment confirmed complete nucleotide conservation among the four isolates, and phylogenetic analysis clustered them within the *Hepatocystis* clade, closely related to *Hepatocystis* spp. ex *Pteropus hypomelanus* from Malaysia. All positive detections were from 2019 samples, suggesting temporal variation in infection or vector activity.

**Conclusion:**

This study provides the first molecular evidence of *Hepatocystis* infection in Indonesian NHPs using fecal DNA, expanding current knowledge of parasite distribution and host range. The successful detection of *Hepatocystis* through non-invasive sampling underscores the potential of fecal-based PCR for wildlife disease surveillance. These findings highlight the importance of integrating molecular diagnostics into conservation and One Health frameworks to monitor zoonotic parasites and understand host–vector–pathogen interactions in natural ecosystems.

## INTRODUCTION

Malaria is a mosquito-borne infectious disease caused by *Plasmodium* hemoparasites of the phylum *Apicomplexa*. These protozoans possess specialized invasion organelles, collectively referred to as the apical complex, which enable them to penetrate red blood cells and hepatocytes. Infection by *Plasmodium* species results in a wide range of clinical manifestations, including fever, anemia, headache, and gastrointestinal disturbances such as diarrhea [1]. Beyond humans, *Plasmodium* infects multiple vertebrate hosts, including birds [2–5], reptiles [6–8], and non-human primates (NHPs) [9–12]. NHPs serve as important natural reservoirs for several zoonotic pathogens, including malaria parasites, particularly in areas where their habitats overlap with human settlements [13]. Human infections with *Plasmodium knowlesi*, a simian parasite naturally found in old-world monkeys such as *Macaca nemestrina* and *Macaca fascicularis*, underscore the increasing risk of zoonotic spillover at the human–wildlife interface [14].

In addition to *Plasmodium*, parasites of the genus *Hepatocystis*, which are phylogenetically related within the family *Plasmodiidae*, also infect NHPs [15, 16]. *Hepatocystis* is transmitted through the bites of *Culicoides* biting midges and has been reported in monkeys, bats, hippopotamuses, and squirrels [17]. Unlike *Plasmodium*, *Hepatocystis* completes its development in the liver, forming merocysts without undergoing asexual replication (schizogony) in the bloodstream. Infections are generally subclinical, lacking the characteristic periodic fever of malaria, but may cause mild anemia and hepatic scarring. Chronic infections can have a subtle impact on host physiology and long-term health [18].

Sulawesi Island, Indonesia, hosts several endemic macaque species, *Macaca nigra*, *Macaca nigrescens*, *Macaca hecki*, *Macaca tonkeana*, *Macaca maura*, *Macaca ochreata*, and *Macaca brunnescens*, that are increasingly threatened by habitat loss and infectious diseases, including malaria-like parasites such as *Plasmodium* and *Hepatocystis* [19, 20]. Because blood sampling from wild primates poses logistical and ethical challenges, this study adopted a non-invasive molecular approach using fecal samples. A previous study by Brown *et al*. [21] has demonstrated that trace amounts of *Plasmodium* DNA can be successfully amplified from fecal material, allowing effective surveillance without animal capture [21]. Building upon this approach, Siregar *et al*. [22] developed a polymerase chain reaction (PCR)-based technique for detecting *Plasmodium* spp. in macaque feces, providing a reliable tool for monitoring wildlife parasites.

Despite Indonesia’s high biodiversity and the coexistence of humans and wildlife across fragmented landscapes, the molecular detection and genetic characterization of malaria-like parasites in NHPs remain poorly documented. Most research on *Plasmodium* and *Hepatocystis* has focused on African and Southeast Asian primate populations, particularly in Malaysia, Myanmar, and Thailand, where zoonotic malaria caused by *P. knowlesi* and related species has been extensively investigated. However, data from Indonesia are scarce, with most studies limited to historical microscopy-based reports from the mid-20^th^ century. Since those early records, no contemporary molecular investigations have been conducted to confirm the presence, diversity, or phylogenetic position of *Hepatocystis* in Indonesian wildlife. Moreover, surveillance efforts have traditionally relied on blood sampling, which presents ethical, logistical, and safety challenges when dealing with wild or protected species. This has led to a critical knowledge gap in understanding the current distribution, host range, and evolutionary relationships of *Hepatocystis* circulating among Indonesian primates. Furthermore, the potential role of these parasites in cross-species transmission dynamics remains unaddressed, despite growing evidence of *Plasmodium*-like infections with zoonotic potential in neighboring regions. Addressing these gaps is essential to improve our understanding of primate health, vector ecology, and pathogen evolution within a One Health framework, linking wildlife conservation and zoonotic disease surveillance.

This study aimed to detect and characterize malaria-like parasites in NHPs from Indonesia using a non-invasive molecular approach based on fecal DNA analysis. Specifically, we sought to (i) screen fecal samples from multiple *Macaca* species and other primates for *Plasmodium* DNA using PCR targeting the mitochondrial *small subunit ribosomal RNA* (*SSU rRNA*) gene, (ii) identify the detected parasite species through DNA sequencing and phylogenetic analysis, and (iii) assess their genetic relationships with previously described *Plasmodium* and *Hepatocystis* isolates from other geographic regions. By applying a fecal-based molecular method, this research aimed to provide the first molecular evidence of *Hepatocystis* infection in Indonesian NHPs, offering new insights into parasite ecology and transmission. The findings are expected to contribute to improved surveillance strategies for malaria-like parasites in wildlife populations and to support the integration of non-invasive pathogen monitoring into One Health-oriented conservation and public health programs in Indonesia.

## MATERIALS AND METHODS

### Ethical approval

All procedures involving non-human primates (NHPs) were reviewed and approved by the Animal Care and Use Ethics Committee of the National Research and Innovation Agency, Indonesia (Approval No. 004/KE.02/SK/1/2025). Fecal sampling was strictly non-invasive; animals were neither captured, restrained, anesthetized, nor experimentally infected for the purposes of this study. Freshly voided feces were collected from the floor of individual or group enclosures during routine husbandry activities, with care taken to minimize disturbance and to avoid cross-contamination between individuals. Permission to access the animals and collect samples at the Tasikoki Wildlife Rescue Center (Manado, North Sulawesi, Indonesia) was obtained from the Ministry of Environment and Forestry and the Natural Resources Conservation Agency (BKSDA) of North Sulawesi, in accordance with national wildlife conservation regulations. All procedures conformed to the National Research and Innovation Agency animal welfare guidelines and internationally recognized principles for the ethical use of animals in research, including the replacement, reduction, and refinement.

### Study duration and location

The study was conducted between March and December 2024 at the Genomics Laboratory, National Research and Innovation Agency, located within the Soekarno Science and Technology Area (KST Soekarno), Cibinong, Bogor, West Java, Indonesia.

### Sample size and collection strategy

Fecal samples were collected from NHPs housed at the Tasikoki Wildlife Rescue Center, Manado, Indonesia, using a convenience sampling approach aimed at detecting malaria-like parasites. A total of 227 freshly voided morning fecal samples were collected, 163 samples in 2019 and 64 samples in 2021.

For individually housed animals, one sample was collected per cage, while for group-housed animals, the number of samples corresponded to the number of individuals. To prevent contamination, only fresh, uncontaminated feces were collected, excluding dried or mixed material.

The collected samples represented a wide range of species, including:

*M. nigra* (n = 106), *M. hecki* (n = 45), *M. nigrescens* (n = 22), *M. tonkeana* (n = 6), *M. fascicularis* (n = 11), *M. nemestrina* (n = 2), *M. maura* (n = 12), *M. brunnescens* (n = 1) as well as other primates: *Nycticebus* spp. (n = 11), *Trachypithecus auratus* (n = 4), *Symphalangus syndactylus* (n = 2), *Hylobates muelleri* (n = 3), and *Hylobates agilis* (n = 2).

### Sample preservation and transport

Each fecal sample was immediately preserved in sterile cryovial tubes (Corning Cryogenic Vials, Corning Inc., USA) containing RNAlater Stabilization Solution (Qiagen) at a 1:1 ratio to stabilize nucleic acids and minimize the effects of PCR inhibitors present in fecal material. Samples were stored in cool boxes with ice packs during field transport to maintain a stable low temperature. Upon arrival at the laboratory, they were transferred to a −20°C freezer for long-term storage.

All specimens are curated under the Structural Biology and Cell Signaling Research Group, Eijkman Research Center for Molecular Biology, National Research and Innovation Agency.

### DNA extraction and quality assessment

Genomic DNA was extracted from the preserved fecal material using the QIAamp Fast DNA Stool Mini Kit (Qiagen) according to the manufacturer’s instructions. DNA yield and purity were determined using a NanoDrop spectrophotometer (Implen GmbH, Munich, Germany; version NPOS 4.2h, build 14900). Only samples with an A260/280 ratio between 1.8 and 2.0 were considered suitable for downstream analysis.

### PCR amplification and controls

Detection of malaria-like parasites was performed through PCR targeting a fragment of the mitochondrial *SSU rRNA* gene specific to *Plasmodium* spp. Each 25 μL PCR reaction mixture contained:

2.5 μL DNA template, 5 μL of 5 × PCR buffer (1.5 mM MgCl_2_; KapaBiosystems, USA), 0.5 μL deoxynucleotide triphosphates (10 mM), 0.25 μL each of forward and reverse primers (40 μM each; Forward: 5′-CAG TGC TCC ATT CAA GGC ATA GA-3′, Reverse: 5′-CCA TTG GAA TGA GAG TTC ACC GT-3′), 16.4 μL nuclease-free water, and 0.1 μL Taq DNA polymerase (0.5 U).

Positive and negative controls were included in each run: *Plasmodium falciparum* (falciparum chloroquine resistant-3 strain) genomic DNA as a positive control and nuclease-free water as a negative control. To ensure reliability, all reactions were performed in triplicate.

Thermal cycling conditions were as follows:

Initial denaturation at 94°C for 5 min; 34 cycles of denaturation at 94°C for 15 s, annealing at 56°C for 15 s, and extension at 72°C for 45 s; with a final extension at 72°C for 5 min. Amplicons were stored at 4°C until further analysis.

### Gel electrophoresis and visualization

PCR products were resolved on 2% agarose gels stained with GelRed (Biotium, USA) and electrophoresed at 80 V for 60 min. DNA bands were visualized using a Uvitec Cambridge gel documentation system (Uvitec Ltd., Cambridge, UK), and fragment sizes were estimated using a ɸX174 DNA–HaeIII Digest marker (Thermo Fisher Scientific, USA). The expected amplicon size for *Plasmodium* spp. was approximately 467 bp.

### Amplicon purification and sequencing

PCR-positive products were purified using the QIAquick PCR purification kit (Qiagen) following the manufacturer’s protocol. Bidirectional Sanger sequencing was performed with the same primer set at the Central Sequencing Laboratory, Soekarno Science and Technology Area, Cibinong Science Center, National Research and Innovation Agency, Bogor, Indonesia.

### Sequence editing and alignment

Raw sequencing chromatograms were reviewed and manually edited using BioEdit v5.0.9 (Ibis Biosciences, USA). Forward and reverse reads were aligned to generate consensus sequences, which were then compared to reference sequences using the basic local alignment search tool-nucleotide (BLASTn) (National Center for Biotechnology Information [NCBI], National Institutes of Health [NIH], Bethesda, Maryland, USA) for species identification. All sequences were aligned using the multiple sequence alignment (MSA) function in BioEdit to evaluate intra-sample similarity and to prepare datasets for phylogenetic reconstruction. Database comparisons were conducted against GenBank (accessed December 11, 2024).

### Phylogenetic and bioinformatics analyses

Phylogenetic trees were constructed using the neighbor-joining (NJ) method implemented in MEGA X v10.2.6 (MEGA Software Team, Temple University, USA), employing 1,000 bootstrap replicates to assess node confidence. The Tamura 3-parameter model was applied for branch length estimation, and pairwise deletion was used to eliminate ambiguous positions. Pairwise genetic distances were also computed in MEGA X to quantify sequence divergence and confirm taxonomic placement.

## RESULTS

### PCR detection of mitochondrial *SSU rRNA* gene fragments

A total of 227 DNA samples extracted from NHP fecal specimens collected in 2019 (n = 163) and 2021 (n = 64) were screened for *Plasmodium* infection using PCR targeting the mitochondrial *SSU rRNA* gene. DNA concentrations ranged from 20 to 282 ng/μL, with purity ratios (A260/280) between 1.9 and 2.0, indicating high-quality DNA suitable for amplification.

Out of all samples tested, 8 samples (3.5%) produced visible DNA bands following agarose gel electrophoresis. The amplicons were approximately 600 bp in size, larger than the expected 467 bp fragment typical of *Plasmodium* spp., suggesting that the amplified DNA originated from non-*Plasmodium* species. The remaining 219 samples (96.5%) showed no visible bands and were considered negative for *Plasmodium*-like DNA.

Based on band clarity, intensity, and purity, four samples were selected for further sequencing and phylogenetic analyses. These were identified as HM-160 (lane 2, *H. muelleri*), MNig-17 (lane 4), MNig-18 (lane 5), and MNig-01 (lane 6), the latter three derived from *M. nigra* individuals. Notably, all positive amplifications were from samples collected in 2019, while no amplifications were detected in the 2021 collection, suggesting temporal variation in parasite detection.

### Molecular identification of *Plasmodium*-like parasites

Species identification was carried out using BLASTn searches against the NCBI GenBank nucleotide database for the four selected isolates (MNig-01, MNig-17, MNig-18, and HM-160). Sequence quality was manually verified, and low-quality bases (~20 bp) at the ends were trimmed before analysis.

The BLAST results (Table 1) revealed that all four isolates shared the highest similarity with *Hepatocystis* spp. (GenBank accession KY653782.1), showing 100% query coverage, E-value = 0, and 99.77%–99.78% identity. These findings indicate that the amplified DNA sequences were more closely related to *Hepatocystis* than to *Plasmodium*, confirming the presence of a malaria-like parasite of the genus *Hepatocystis* in the examined NHPs.

**Table 1:** Basic local alignment search tool-nucleotide analysis showed that all 4 amplified samples (MNig-01, MNig-17, MNig-18, and HM-160) were most similar to *Hepatocystis* spp. (KY653782.1), rather than *Plasmodium* spp., suggesting the presence of *Hepatocystis* DNA in these samples.

Sample code	Host	Closest match (National Center for Biotechnology Information accession No.)	Query coverage (%)	E-value	Percent identities
MNig-01	*Macaca nigra*	*Hepatocystis* spp. (KY653782.1)	100	0	99.77
MNig-17	*Macaca nigra*	*Hepatocystis* spp. (KY653782.1)	100	0	99.78
MNig-18	*Macaca nigra*	*Hepatocystis* spp. (KY653782.1)	100	0	99.78
HM-160	*Hylobates muelleri*	*Hepatocystis* spp. (KY653782.1)	100	0	99.78

**Figure 1:**
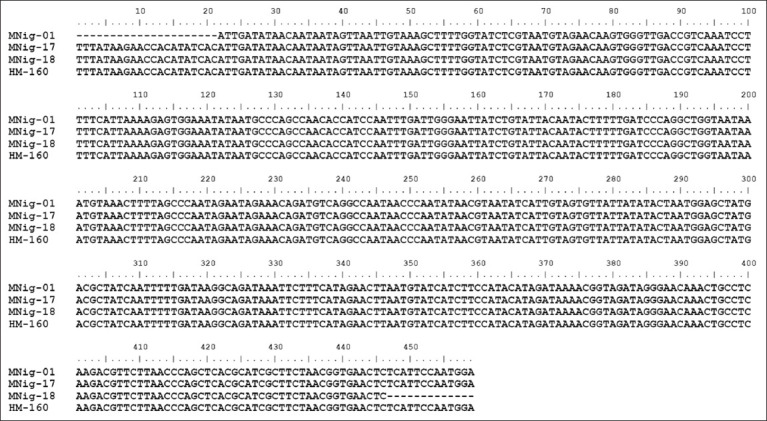
Multiple sequence alignment shows the identical small subunit ribosomal RNA gene in the analyzed samples. The alignment of MNig-01, MNig-17, MNig-18, and HM-160 reveals complete conservation, suggesting that they are derived from the same Hepatocystis species or closely related strains.

To assess intraspecific variation, MSA was performed on the four isolates (Figure 1). The alignment revealed complete nucleotide conservation across the amplified mitochondrial *SSU rRNA* region, with no detectable polymorphisms among samples. This high sequence uniformity suggests that the isolates represent the same species or closely related strains within a single evolutionary lineage of *Hepatocystis*.

### Phylogenetic analysis and evolutionary relationships

**Figure 2:**
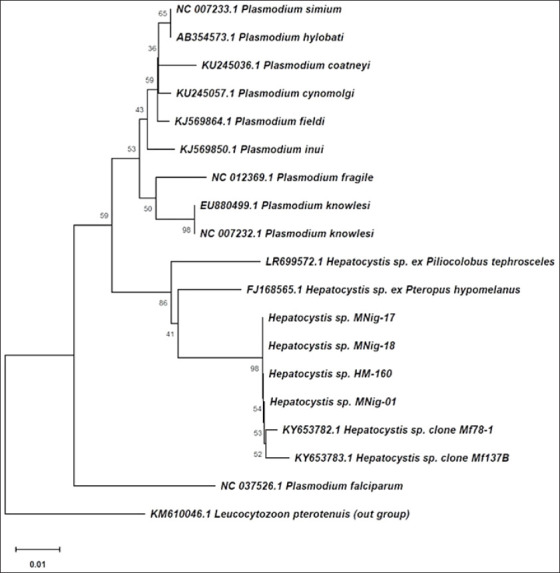
Phylogenetic tree based on mitochondrial small subunit ribosomal RNA gene sequences with 1000 bootstrap replicates shows MNig-01, MNig-17, MNig-18, and HM-160 clustering in the Hepatocystis clade, closely related to KY653782.1 and KY653783.1.

Phylogenetic reconstruction based on mitochondrial *SSU rRNA* sequences (Figure 2) demonstrated two well-defined clades within the family *Plasmodiidae*: one representing *Plasmodium* spp. and the other *Hepatocystis* spp. All four study isolates, MNig-01, MNig-17, MNig-18, and HM-160, clustered firmly within the *Hepatocystis* clade, confirming their taxonomic placement.

The p-distance analysis (Table 2) revealed no genetic divergence (0.000) among the four sequences, supporting their classification as a single species or strain. The isolates showed the closest genetic relationship with *Hepatocystis* spp. (KY653782.1), followed by *Hepatocystis* spp. (KY653783.1); both were previously reported from *M. fascicularis* in Southeast Asia [23].

**Table 2:** Pairwise p-distance between the analyzed samples and reference sequences. All 4 analyzed sequences (MNig-01, MNig-17, MNig-18, and HM-160) exhibited a p-distance of 0.000 (0% divergence), indicating complete genetic identity. The smallest genetic distance was observed for *Hepatocystis* spp. (KY653782.1), with a p-distance of 0.003 (0.3% divergence).

No.	Sample code	1	2	3	4	5	6	7	8	9	10	11	12	13	14	15	16	17	18	19
1.	MNig-17																			
2.	MNig-18	0.000																		
3.	HM-160	0.000	0.000																	
4.	MNig-01	0.000	0.000	0.000																
5.	KY653782.1	0.003	0.003	0.003	0.003															
6.	KY653783.1	0.005	0.005	0.005	0.005	0.008														
7.	FJ168565.1	0.032	0.032	0.032	0.032	0.035	0.037													
8.	LR699572.1	0.041	0.041	0.041	0.041	0.043	0.041	0.032												
9.	KJ569864.1	0.042	0.042	0.042	0.042	0.045	0.048	0.037	0.042											
10.	NC 007233.1	0.042	0.042	0.042	0.042	0.045	0.048	0.037	0.042	0.006										
11.	AB354573.1	0.042	0.042	0.042	0.042	0.045	0.048	0.037	0.042	0.006	0.000									
12.	KU245057.1	0.045	0.045	0.045	0.045	0.048	0.050	0.039	0.045	0.006	0.006	0.006								
13.	KJ569850.1	0.045	0.045	0.045	0.045	0.048	0.050	0.045	0.045	0.011	0.011	0.011	0.011							
14.	KU245036.1	0.050	0.050	0.050	0.050	0.053	0.056	0.045	0.050	0.011	0.011	0.011	0.011	0.017						
15.	NC 012369.1	0.051	0.051	0.051	0.051	0.054	0.056	0.048	0.051	0.020	0.022	0.022	0.022	0.022	0.025					
16.	EU880499.1	0.053	0.053	0.053	0.053	0.056	0.059	0.051	0.053	0.019	0.019	0.019	0.017	0.019	0.025	0.020				
17.	NC 007232.1	0.053	0.053	0.053	0.053	0.056	0.059	0.051	0.053	0.019	0.019	0.019	0.017	0.019	0.025	0.020	0.000			
18.	NC 037526.1	0.059	0.059	0.059	0.059	0.062	0.065	0.056	0.059	0.051	0.051	0.051	0.051	0.054	0.054	0.060	0.054	0.054		
19.	KM610046.1	0.087	0.087	0.087	0.087	0.090	0.092	0.084	0.084	0.064	0.064	0.064	0.064	0.059	0.070	0.068	0.059	0.059	0.071	

Within the *Hepatocystis* lineage, the study samples formed a distinct subclade closely related to *Hepatocystis* spp. ex *Pteropus hypomelanus* (FJ168565.1) from Malaysian fruit bats and *Hepatocystis* spp. ex *Piliocolobus tephrosceles* (LR699572.1) from Ugandan red colobus monkeys. These relationships indicate minor genetic variation but strong evolutionary coherence within the genus.

Additional reference sequences, including *Plasmodium simium* (NC007233.1), *Plasmodium cynomolgi* (KU245057.1), *P. knowlesi* (EU880499.1), and *P. falciparum* (NC037526.1), clustered within the *Plasmodium* clade and were genetically distinct from the *Hepatocystis* isolates. Notably, *P. falciparum* formed a separate branch, highlighting its divergent evolutionary trajectory within the family.

The NJ phylogenetic tree, rooted using *Leucocytozoon pterotenuis* as an outgroup, confirmed that all four analyzed samples belong to the genus *Hepatocystis*, a hemosporidian parasite infecting primates and bats. These findings provide robust molecular evidence for the taxonomic and evolutionary placement of the detected parasites within *Hepatocystis* and highlight their close relationship to Southeast Asian lineages.

## DISCUSSION

### Diagnostic PCR targeting the mitochondrial *SSU rRNA* gene of Plasmodium spp.

#### Principle and target gene selection

PCR is a highly sensitive and specific molecular technique widely used to detect target DNA within biological samples. In this study, PCR was employed to identify the presence of parasitic DNA in fecal samples from NHPs, specifically targeting the mitochondrial *SSU rRNA* gene region. The mitochondrial genome was selected as the amplification target due to its high copy number per cell, increased resistance to environmental degradation, and relatively low mutation rate compared with nuclear DNA, making it particularly suitable for molecular analysis of degraded samples, such as fecal or preserved tissues [24–26]. Primers were designed to amplify the *SSU rRNA* gene located in the mitochondrial genome of *Plasmodium*. This gene contains highly conserved regions across different species and possesses sufficient variability to enable reliable species differentiation [22, 27].

#### Non-invasive detection using fecal DNA

In addition to the target gene, the sample type plays a critical role in molecular detection. Although the detection of *Plasmodium* often relies on blood samples as the primary DNA source, fecal-based molecular diagnostics have emerged as a reliable non-invasive alternative. A previous study by Siregar *et al*. [22] demonstrated that the sensitivity of fecal and blood samples is comparable, supporting the utility of fecal DNA for parasite detection.

#### PCR screening results

**Figure 3:**
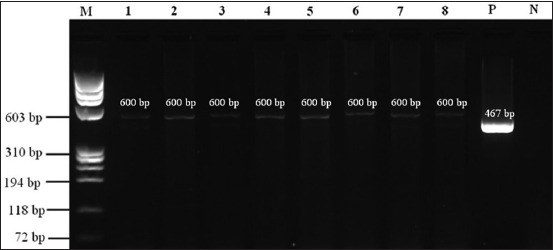
Polymerase chain reaction products of the mitochondrial small subunit *rRNA* gene on 2% agarose gel. Bands (~600 bp) observed in some samples differ from the expected 467 bp for *Plasmodium*, suggesting possible non-*Plasmodium* mitochondrial amplification. Lane M: DNA size marker (ladder) a ɸX174 DNA–*Hae* III digest; Lane 1: MNig-22; Lane 2: HM-160; Lane 3: MNig-21; Lane 4: MNig-17; Lane 5: MNig-18; Lane 6: MNig-01; Lane 7: MNig-05; Lane 8: MNig-10; Lane P: Positive control (*Plasmodium falciparum* falciparum chloroquine resistant-3 (laboratory strain)); Lane N: Negative control.

Of the total samples, 8 (3.5%) exhibited detectable DNA bands (Figure 3). The yield and purity of genomic DNA extracted from fecal samples were measured using a NanoDrop spectrophotometer, with concentrations ranging from 20 to 282 ng/μL and A260/280 ratios between 1.9 and 2.0, indicating that the extracted DNA was of high purity and suitability for downstream molecular analyses [28]. The amplified bands were approximately 600 bp in size, which was larger than the expected 467 bp amplicon corresponding to the *Plasmodium* mitochondrial *SSU rRNA* gene. This unexpected size discrepancy suggests that the samples likely did not contain *Plasmodium* DNA, necessitating further sequence-based analysis to accurately identify the amplified taxa. Sequencing and phylogenetic analyses were proposed for this purpose, with sequence similarity confirmed against public databases such as GenBank. Among the 8 samples that showed DNA bands in the PCR process, 4 samples (MNig-01, MNig-17, MNig-18, and HM-160) were selected for further processing, including DNA purification and sequencing. Selection criteria were based on band clarity, amplicon quality, and DNA purity to ensure the reliability of sequencing results and minimize artifacts due to degraded or low-concentration DNA. Sequence data were analyzed using BioEdit software and BLAST searches using the NCBI platform.

#### Factors influencing PCR success

Several factors were identified as critical to the successful amplification of mitochondrial DNA through PCR. First, the quality and quantity of DNA templates are crucial, especially due to the presence of PCR inhibitors in fecal material, such as complex polysaccharides, bile salts, bilirubin, and hemoglobin degradation products, which can interfere with enzymatic reactions [29, 30]. Second, primer specificity and efficiency are essential. Primers must anneal to conserved regions while retaining sufficient variability to distinguish between closely related species [31–33]. Third, optimizing PCR conditions, such as annealing temperature, MgCl_2_ concentration, and number of cycles, is crucial for achieving robust amplification [34–36].

#### Significance of Fecal-based PCR in wildlife health surveillance

This study highlights the utility of mitochondrial *SSU rRNA* gene amplification from fecal samples as a reliable indicator of parasitic presence. PCR-based DNA detection is not only crucial for molecular identification and phylogenetic inference but also holds significant value in epidemiological surveillance and conservation biology. Non-invasive molecular approaches, such as those utilizing fecal samples, offer powerful alternatives for monitoring infection status and population health in wild primates without the need for capture or direct handling, thereby minimizing disturbance and avoiding stress or harm to the animals. Previous studies [1, 22, 37, 38] have demonstrated the success of fecal DNA analysis for species identification, population monitoring, and pathogen detection, reinforcing its relevance in conservation genetics and ecological studies.

### Molecular identification and species determination of *Plasmodium*-like parasites based on mitochondrial *SSU rRNA* gene sequences

#### BLAST-based molecular identification

Molecular-based species identification has become a pivotal approach across various biological disciplines, including taxonomy, ecology, and epidemiology of parasitic diseases. Unlike morphological methods, which are often limited in accuracy, particularly in cases of degraded specimens or cryptic species, molecular approaches enable species recognition through genetic information encoded in DNA [39, 40]. In this study, molecular identification was conducted using DNA sequence analysis through BLAST, hosted by the NCBI. This technique facilitates high-accuracy species identification by comparing the query sequence with reference sequences in the GenBank database [41].

#### Sequence similarity and identification results

BLAST analysis (Table 1) of the 4 tested samples, MNig-01, MNig-17, MNig-18, and HM-160, revealed exceptionally high sequence similarity to *Hepatocystis* spp., with identity percentages of 99.77%, 99.78%, 99.78%, and 99.78%, respectively. These findings strongly support the conclusion that all 4 isolates represent the same species, identified as *Hepatocystis* spp. (GenBank accession no. KY653782.1). A genetic identity of ≥97% indicates that the samples belong to the same species, whereas similarity between 94% and 97% suggests that they are related at the genus level. The data clearly show that all 4 isolates belong to the same species [42–44]. In addition, all samples yielded an E-value of 0 in the BLAST results, indicating that the observed sequence similarity between the queries and reference sequences is not a product of random chance but rather reflects true biological homology [45, 46]. Furthermore, samples MNig-01, MNig-17, MNig-18, and HM-160 exhibited 100% query coverage. These findings provide robust statistical and biological evidence supporting the accuracy and significance of molecular identification.

#### MSA and temporal pattern

An MSA was performed to further confirm that the four samples represent a single species. The alignment (Figure 1) showed complete sequence identity across all samples, supporting the conclusion that they belong to the same *Hepatocystis* lineage and are derived from wild primate hosts, based on the reference sequence KY653782.1 from *M. fascicularis* [23]. These molecular results provide strong evidence that all positive samples represent a single parasite strain circulating within the captive primate population at that time. Interestingly, DNA bands were detected only in the 2019 collection, with 4 samples confirmed as *Hepatocystis*, whereas no amplification was obtained from the 2021 collection. This temporal pattern likely reflects natural fluctuations in parasite transmission dynamics, which could be influenced by differences in sample size, environmental factors (e.g., temperature and rainfall) affecting vector activity [47], and the health status of the host population. The absence of positive samples in 2021 does not necessarily indicate the disappearance of the parasite, but rather temporal variation in transmission. These findings underscore the importance of long-term monitoring to achieve a more accurate understanding of the dynamics of infection.

#### Epidemiological significance and One Health implications

The molecular approach employed in this study provides valuable insights into the health status of NHP populations and their potential role as natural reservoirs of *Hepatocystis*, a parasite closely related to *Plasmodium* [48, 49]. Moreover, it emphasizes the importance of considering human–wildlife interactions. This is supported by evidence that *Anopheles letifer* in Kalimantan has been identified to carry malaria sporozoites from NHPs, indicating the potential for cross-species transmission in areas where habitats and human activities overlap [50]. The detection of *Hepatocystis* in captive primates further strengthens the possibility that NHPs may serve as previously unrecognized reservoirs, particularly in habitats adjacent to human activities, where competent vectors are present and cross-species contact occurs.

### Taxonomic context and ecological distribution of *Hepatocystis*

#### Taxonomy and host range

Understanding the taxonomic position, host range, and previous records of *Hepatocystis* in Indonesia is crucial for situating these findings within a broader biological and historical context. *Hepatocystis* is a genus of protozoan parasites closely related to *Plasmodium*, the causative agent of malaria. Both genera belong to the phylum *Apicomplexa*, order *Coccidia*, and family *Plasmodiidae* [51, 52]. *Hepatocystis* species infect a variety of mammalian hosts, particularly primates, bats, squirrels, and ungulates across Africa, Asia, and Australia. Transmission occurs through the bite of minute hematophagous insects from the genus *Culicoides* [17].

#### Historical reports in Indonesia

The presence of *Hepatocystis* has been documented in several wild mammal species, particularly NHPs and fruit bats, in Indonesia. One of the earliest records, dating back to 1963, was the identification of *Hepatocystis semnopithecus* in 6 out of 14 *M. fascicularis* (previously classified as *M. irus*) captured near Bogor, West Java. The infection was presumed to be acquired naturally due to the extended incubation period, making transmission during laboratory quarantine unlikely [53]. Additional reports include the detection of *Hepatocystis* in *Pongo pygmaeus* from South Kalimantan in 1978 [54] and *Hepatocystis pteropi* infection in fruit bats (*Cynopterus brachyotis* and *Cynopterus horsfieldi*) in the same region, with prevalences of 39% and 29%, respectively [55]. Since these initial reports, no subsequent scientific publications have documented the occurrence of *Hepatocystis* in Indonesia.

#### Vector ecology in Sulawesi

Given that *Culicoides* biting midges transmit *Hepatocystis* [17], understanding the ecological suitability of Sulawesi for these vectors is crucial. The island’s tropical climate, consistently high humidity, and extensive forested habitats provide favorable conditions for *Culicoides* breeding and activity [56, 57], which may facilitate parasite transmission among primate populations. Although there is limited direct evidence of *Culicoides*-mediated transmission in Indonesia, these ecological characteristics suggest that local primates may be exposed to vector bites.

### Pathobiology and conservation implications

Although *Hepatocystis* is a hemoparasite phylogenetically related to *Plasmodium*, infections in vertebrate hosts are typically asymptomatic or associated with only mild clinical signs. Documented pathological outcomes remain scarce; however, anemia has been reported in some instances, likely resulting from merozoite invasion combined with the host’s immune response. In contrast to *Plasmodium*, *Hepatocystis* does not undergo asexual replication (schizogony) in the bloodstream and therefore does not trigger the characteristic febrile cycles of malaria. In general, only gametocytes are present in the peripheral circulation, and these forms do not stimulate a strong proinflammatory cytokine response, thereby preventing the cytokine storm that is commonly responsible for fever and systemic inflammation during *Plasmodium* infections. *Hepatocystis* completes its merogony (asexual development) exclusively in the liver, where it produces large, macroscopically visible merocystis. As the host attempts to contain parasite replication, this hepatic stage can provoke localized immune responses, including leukocyte infiltration and tissue inflammation [17].

Parasitic infections can have subtle yet significant consequences for wildlife conservation, even when overt clinical symptoms are absent, particularly in threatened primate populations. Although no direct studies have linked *Hepatocystis* infection to changes in host behavior or physiology, similar patterns have been observed in other parasites. For instance, *Trichuris*-infected red colobus monkeys exhibit longer resting periods and reduced engagement in energetically demanding activities during parasite shedding, suggesting that subclinical infections can impose physiological and behavioral costs [58]. In addition, helminth infections in primates inhabiting fragmented forests in the Udzungwa Mountains have been linked to shifts in gut microbiota composition, reflecting the complex interactions between parasites, host health, and environmental pressures [59]. These insights emphasize that even seemingly mild or latent infections have conservation significance. Consequently, the systematic surveillance of *Hepatocystis* is essential for understanding host–parasite dynamics, zoonotic potential, and the ecological role of local vectors such as *Culicoides* spp.

### Novelty and broader implications

To the best of our knowledge, this is the first study to detect *Hepatocystis* from fecal samples of NHPs in Indonesia. This novel finding has several important implications. First, it demonstrates the utility of non-invasive surveillance for hemoparasites in wildlife, enabling pathogen monitoring without the need for capture or blood sampling [48]. Second, the detection of *Hepatocystis* in fecal material contributes to One Health–oriented zoonotic risk assessment because fecal monitoring can reveal parasite presence across human-wildlife interfaces and inform spillover risk mitigation [60]. Third, from a conservation perspective, documenting *Hepatocystis* circulation among primate populations highlights the need to integrate parasitological surveillance into management plans for threatened species because even apparently subclinical infections may impose physiological or behavioral costs that reduce fitness in disturbed or fragmented habitats [58, 59]. Altogether, these results underscore the value of broadening routine wildlife health monitoring to include molecular screening of non-invasive samples. We recommend longitudinal fecal surveillance and targeted vector investigations to clarify transmission dynamics and conservation implications.

### Phylogenetic analysis based on the mitochondrial *SSU rRNA* gene

#### Tree construction and analytical approach

Phylogenetic reconstruction was performed using sequence data from the mitochondrial *SSU rRNA* gene. The resulting phylogenetic tree, constructed using the NJ method [61], illustrates the evolutionary relationships among species within the *Plasmodiidae* family. Taxa are grouped according to genetic similarity, whereby species sharing a more recent common ancestor cluster more closely on the tree [62]. The phylogenetic analysis employed *L. pterotenuis* as an outgroup, a genus of parasitic alveolate belonging to the phylum *Apicomplexa* (which also includes malaria parasites) [63]. Overall, the topology revealed two major clades (Figure 2). The first comprises various *Plasmodium* species, such as *P. simium*, *P. cynomolgi*, *P. knowlesi*, and others known to infect NHPs. The second clade consists solely of *Hepatocystis* isolates, forming a distinct and genetically divergent branch from *Plasmodium*. Although both genera belong to the same family (*Plasmodiidae*), *Hepatocystis* exhibits substantial evolutionary separation and phylogenetic distinctness from *Plasmodium* [18]. An additional isolated branch is represented by *P. falciparum*, the primary human malaria pathogen. Its position, separate from both major clades, suggests a more distant evolutionary lineage, underscoring its unique phylogenetic status among hemosporidian parasites.

#### Placement of study isolates

The four primary samples in this study, *Hepatocystis* spp. from MNig-01, MNig-17, and MNig-18 (hosted by *M. nigra*) and HM-160 (hosted by *H. muelleri*), formed a single monophyletic clade arranged in a linear pattern. This topology indicates a very close evolutionary relationship among the samples, suggesting that they likely originated from a common ancestor. It is highly probable that all four samples belong to the same *Hepatocystis* species or to distinct but closely related species within the same taxonomic group. Their phylogenetic positioning also reflects a low level of genetic divergence, implying that only minimal nucleotide changes or mutations have occurred [64, 65]. These findings are further supported by BLAST analysis (Table 1), which shows >99% sequence similarity to *Hepatocystis* spp. clone Mf78-1 (KY653782.1), as well as by MSA results (Figure 1), which revealed complete sequence identity across the full alignment with no observed mutations. Moreover, these four samples clustered closely with two reference isolates, *Hepatocystis* spp. clones Mf78-1 (KY653782.1) and Mf137B (KY653783.1), both previously identified from long-tailed macaques (*M. fascicularis*) [23]. The six sequences formed a subclade within the larger *Hepatocystis* clade, suggesting that the samples may have been obtained from hosts with similar ecological backgrounds or from nearby geographic areas. In addition, the similarity in genetic sequences might be due to shared evolutionary history or exposure to the same selective environmental pressures [66, 67]. Meanwhile, two other isolates of the *Hepatocystis* genus, namely *Hepatocystis* spp. ex *P. hypomelanus* from a Malaysian fruit bat (FJ168565.1) [68] and *Hepatocystis* spp. ex *P. tephrosceles* from a Ugandan red colobus monkey (LR699572.1) [18], were positioned further away from both the four study samples and the reference isolates (KY653782.1 and KY653783.1). This distance indicates greater genetic diversity in *Hepatocystis* species from different hosts, suggesting that host species play an important role in the evolution and speciation of *Hepatocystis* [69].

#### Genetic distance analysis

Genetic distances across 19 mitochondrial *SSU rRNA* gene sequences were further quantified using p-distance analysis (Table 2). The four analyzed samples (MNig-01, MNig-17, MNig-18, and HM-160) exhibited a p-distance of 0.000 (0% divergence) among themselves, indicating that the sequences were identical with no detectable nucleotide differences. This provides strong evidence for their classification within the same taxon or a very closely related lineage. These samples also displayed minimal divergence from *Hepatocystis* spp. KY653782.1 and KY653783.1 [23], with p-distance values ranging from 0.003 to 0.005 (0.3%–0.5% divergence), further supporting their inclusion within the *Hepatocystis* group. In contrast, comparisons with other *Hepatocystis* isolates, such as FJ168565.1 from a Malaysian fruit bat [68] and LR699572.1 from *Piliocolobus* [18], yielded higher p-distances (0.032–0.041), corresponding to 3.2%–4.1% divergence, reflecting greater evolutionary divergence within the genus. The *Plasmodium* species infecting NHPs, including *P. simium* (NC 007233.1) [70], *Plasmodium hylobati* (AB354573.1), *P. cynomolgi* (KU245057.1) [71], *Plasmodium fieldi* (KJ569864.1), *Plasmodium coatneyi* (KU245036.1), *Plasmodium inui* (KJ569850.1) [72], *Plasmodium fragile* (NC 012369.1) [70], and *P. knowlesi* (EU880499.1; NC 007232.1) exhibited p-distances in the range of 0.042–0.053 (4.2%–5.3% divergence) compared to *Hepatocystis*, indicating a moderate degree of phylogenetic separation. Notably, *P. falciparum* (NC037526.1) [73] showed the highest p-distance (0.059; 5.9% divergence), reinforcing its status as the most evolutionarily divergent species in the dataset. This finding aligns with prior evidence suggesting that *P. falciparum*, a human-specific parasite, has undergone an independent evolutionary trajectory distinct from other *Plasmodium* lineages [73–75].

### Documented Hepatocystis occurrence in Asia, Africa, and Australasia

#### Geographical distribution and host range

*Hepatocystis* has been widely reported in tropical and subtropical regions, with infections documented in a variety of mammalian hosts, particularly primates, bats, and squirrels. In Africa, Uganda stands out as a key location where *Hepatocystis* has been identified in wild primates, such as *Papio anubis*, *Cercopithecus ascanius*, and *Colobus guereza* [76]. In addition, a study conducted in Cameroon reported a high prevalence of *Hepatocystis* in fruit bats (*Epomophorus* spp.), highlighting the potential role of bats as alternative reservoir hosts [77].

#### Occurrence in Asia

*Hepatocystis* infections have been reported in several Asian countries, with a notable concentration in Southeast Asia. The early records of bat infections in this region were based on microscopic examination. These include reports of *H. pteropi* in *Pteropus* bats in Malaysia, undescribed *Hepatocystis* species in *Hipposideros* bats in Malaysia, and additional species in *C. brachyotis* and *C. horsfieldi* in Indonesia [55, 78, 79]. Molecular studies have since expanded our understanding of *Hepatocystis* diversity in Southeast Asian bats, with sequence data reported from Cambodia, Malaysia, Singapore, and Thailand [69, 80–82]. *Hepatocystis* infections have also been recorded in primates, particularly in macaques. Infections have been identified in species from northern Myanmar near China’s Yunnan Province [16]. Furthermore, in 1963, Eyles and Warren reported *Hepatocystis* infections in *M. irus*, now recognized as *M. fascicularis*, on the island of Java, Indonesia [53].

#### Occurrence in Australasia

Although *Hepatocystis* has not been reported in primates in Australia, infections have been detected in *Pteropus* bats. Prevalence varied by species and geographic region, with low rates observed in *Pteropus poliocephalus* and higher rates in *Pteropus alecto* and *Pteropus scapulatus*. Specifically, the prevalence of *P. poliocephalus* reached 15% in central Queensland and New South Wales, while dropping to only 1% in the southernmost regions of its range [83, 84].

#### Ecological correlates and research needs

The geographical distribution of *Hepatocystis* is closely related to the presence of its vector, *Culicoides* spp., which thrives in moist environments rich in organic matter. As such, tropical countries with intact forest habitats constitute major hotspots for parasites. Expanded research in Southeast Asia and Central Africa is needed to better understand the zoonotic potential and ecological dynamics of the parasite. Table 3 [14, 16, 18, 48, 52–55, 60, 69, 76, 77, 80, 83–106] presents a detailed summary of *Hepatocystis* distribution across countries.

**Table 3:** Geographic distribution of *Hepatocystis* infections across various hosts and continents, including reported host species and supporting literature.

Continent	Country of occurrence	Host species	References
Asia	Singapura	*Cynopterus brachyotis*	[69]
	Indonesia	*Pongo pygmaeus*	[54]
		*Macaca irus (now known as Macaca fascicularis)*	[53]
		*Cynopterus brachyotis Cynopterus horsfieldii*	[55]
	Thailand	*Hipposideros larvatus Hipposideros bicolor Hipposideros armiger Hipposideros lekaguli Rhinolophus malayanus Rhinolophus thomasi Rhinolophus pearsonii Craseonycteris thonglongyai Cynopterus brachyotis*	[80]
		*Hylobates concolor*	[85]
		*Macaca fascicularis*	[86]
		*Macaca fascicularis aurea*	[87]
		*Macaca fascicularis Macaca nemestrina*	[14, 88]
	Malaysia	*Pteropus hypomelanus*	[60]
		*Presbytis aygula*	[89]
		*Rattus tanezumi*	[90]
	Myanmar	*Macaca species*	[16]
	China (Yunnan)	*Macaca mulatta*	[91]
Africa	Cameroon	*Epomophorus pusillus*	[77]
		*Cercopithecus nictitans*	[92]
	Tanzania	*Chlorocebus pygerythrus*	[52]
		*Papio hamadryas s. l.*	[93]
	Nigeria	*Epomophorus* spp. *Micropteropus pusillus Rousettus aegyptiacus*	[94]
		*Eidolon helvum*	[95]
	Uganda	*Piliocolobus tephrosceles*	[18]
		*Colobus guereza Papio anubis Procolobus rufomitratus Cercopithecus ascanius*	[76]
		*Pan troglodytes*	[48]
		*Epomops franqueti Myonycteris torquata*	[96]
	Sudan	*Epomophorus Epomops Hypsignathus Micropteropus Hipposideros*	[97]
	Kenya	*Cercopithecus aethiops*	[98]
	Burkina Faso	*Epomophorus gambianus Epomophorus pusillus*	[99]
	Republic of the Congo	*Miniopterus minor Rhinolophus* spp.	[100]
		*Hypsignathus monstrosus*	[101]
	Gabon	*Epomops franqueti*	[102]
		*Hypsignathus monstrosus*	[103, 104]
		*Cercopithecus cephus Mandrillus sphinx Miopithecus talapoin*	[105]
	Côte d’Ivoire Guinea Liberia	*Epomophorus gambianus Epomops buettikoferi Hypsignathus monstrosus Micropteropus pusillus Myonycteris leptodon Nanonycteris veldkampii*	[106]
Australia	Australia	*Pteropus poliocephalus Pteropus alecto Pteropus scapulatus*	[83, 84]

## CONCLUSION

This study represents the first molecular detection of *Hepatocystis* spp. from fecal samples of NHPs in Indonesia, providing novel insights into the occurrence, phylogenetic position, and potential epidemiological role of this parasite within the *Plasmodiidae*. A total of 227 fecal DNA samples were analyzed using PCR targeting the mitochondrial *SSU rRNA* gene, revealing 8 (3.5%) positive amplifications. Sequencing and BLAST analysis confirmed that four representative isolates, MNig-01, MNig-17, MNig-18, and HM-160, shared >99.7% nucleotide identity with *Hepatocystis* spp. (KY653782.1), forming a distinct monophyletic clade in the phylogenetic tree. No *Plasmodium* DNA was detected, and the amplified fragments (approximately 600 bp) confirmed the presence of *Hepatocystis*-like sequences in Sulawesi primates.

These findings validate the use of non-invasive molecular surveillance based on fecal DNA as a powerful diagnostic tool for hemoparasites in wildlife. This approach minimizes animal stress, eliminates the need for capture or blood sampling, and supports long-term health monitoring of vulnerable populations, while contributing to One Health risk assessment by identifying potential zoonotic interfaces where competent vectors (*Culicoides* spp.) are present. The study’s strength lies in combining molecular diagnostics, sequencing verification, and phylogenetic reconstruction to enable reliable identification of *Hepatocystis* from degraded fecal DNA.

Nevertheless, the limited number of positive samples, restricted sampling period (2019–2021), and absence of vector surveillance constrain the broader generalization of infection prevalence. Environmental DNA degradation and the presence of PCR inhibitors may also have affected detection sensitivity. Future research should focus on extended longitudinal sampling, vector identification, and comparative genomic analysis to better understand transmission dynamics, host–parasite co-evolution, and ecological drivers of *Hepatocystis* distribution.

Overall, this work expands the current understanding of primate hemoparasites in Indonesia, underscores the value of fecal-based molecular monitoring in wildlife disease surveillance, and highlights the importance of integrating non-invasive parasitological assessments into conservation and One Health frameworks to safeguard both primate biodiversity and public health.

## DATA AVAILABILITY

The supplementary data can be made available from the corresponding author upon request.

## AUTHORS’ CONTRIBUTIONS

FH: Experimental work, molecular analysis, and drafted and edited the manuscript. JES: Conceptualization, sampling, experimental work, molecular analysis, and drafted and edited the manuscript. NEP: Sampling, experimental work, and reviewed and edited the manuscript. AFMR: Sampling, experimental work, and reviewed and edited the manuscript. WAA: Experimental work and reviewed and edited the manuscript. IMA: Conceptualization and drafted and edited the manuscript. WK: Conceptualization and reviewed and edited the manuscript. All authors have read and approved the final manuscript.
